# Accurate Estimation of Diffusion Coefficients and their Uncertainties from Computer Simulation

**DOI:** 10.1021/acs.jctc.4c01249

**Published:** 2024-12-30

**Authors:** Andrew R. McCluskey, Samuel W. Coles, Benjamin J. Morgan

**Affiliations:** † Centre for Computational Chemistry, School of Chemistry, University of Bristol, Cantock’s Close, Bristol BS8 1TS, U.K.; ‡ European Spallation Source ERIC, Data Management and Software Centre, Asmussens Allé 305, DK-2800 Kongens Lyngby, Denmark; § Diamond Light Source, Harwell Campus, Didcot OX11 0DE, U.K.; ∥ Department of Chemistry, University of Bath, Claverton Down, Bath BA2 7AY, U.K.; ⊥ The Faraday Institution, Quad One, Harwell Science and Innovation Campus, Didcot OX11 0RA, U.K.

## Abstract

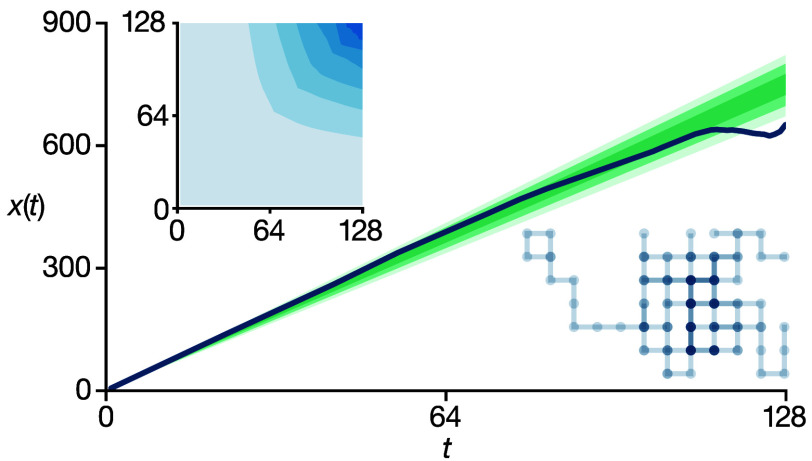

Self-diffusion coefficients, D*, are routinely estimated from molecular dynamics simulations by fitting a linear model to the observed mean squared displacements (MSDs) of mobile species. MSDs derived from simulations exhibit statistical noise that causes uncertainty in the resulting estimate of D*. An optimal scheme for estimating D* minimizes this uncertainty, i.e., it will have high statistical efficiency, and also gives an accurate estimate of the uncertainty itself. We present a scheme for estimating D* from a single simulation trajectory with a high statistical efficiency and accurately estimating the uncertainty in the predicted value. The statistical distribution of MSDs observable from a given simulation is modeled as a multivariate normal distribution using an analytical covariance matrix for an equivalent system of freely diffusing particles, which we parametrize from the available simulation data. We use Bayesian regression to sample the distribution of linear models that are compatible with this multivariate normal distribution to obtain a statistically efficient estimate of D* and an accurate estimate of the associated statistical uncertainty.

## I Introduction

Mass transport is a fundamental physical process that is central to our understanding of fluids^1−3^ and plays a critical role in biochemical systems^4,5^ and solid-state devices, such as batteries, fuel cells, and chemical sensors.^6−8^ Molecular dynamics simulations are widely used to study microscopic transport processes, as they give direct insight into atomic-scale transport mechanisms and can be used to calculate macroscopic transport coefficients.^9−14^ These transport coefficients are formally defined in terms of ensemble averages. Dynamical simulations, however, sample the full ensemble space stochastically, and parameters derived from simulation data therefore are estimates of the true parameter of interest. The statistical uncertainty associated with such estimates depends on the details of the simulation—e.g., size and time scale—and on the choice of estimation method. An optimal estimation method will minimize the uncertainty in the computed quantity, i.e., it will have high statistical efficiency and will also allow this uncertainty to be accurately estimated.

One commonly used parameter for quantifying atomic-scale mass transport is the self-diffusion coefficient, D*, which describes diffusion in the absence of a chemical potential gradient. D* is related to the ensemble-average mean squared displacement (MSD), ⟨Δ**r**(*t*)^2^⟩, via the Einstein relation,^15,16^
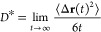
1where Δ**r**(*t*) is the displacement of a diffusing particle in the time interval *t*. Because numerical simulations are finite in time and space, MSDs obtained from simulation data always differ from the true ensemble-average MSD. One can, however, compute an estimate of the self-diffusion coefficient, D̂*, by fitting a linear model to the observed MSD and using the gradient of this fitted model in place of ⟨Δ**r**(*t*)^2^⟩/*t* in eq 1.^17−20^

The simplest approach to fitting a linear model to MSD data from simulation is ordinary least-squares regression (OLS). OLS gives analytical expressions for the “best fit” regression coefficients (the slope and intercept) and their respective uncertainties, making it easy to implement and quick to perform. OLS, however, is appropriate only for data that are both statistically independent and identically distributed. Neither of these conditions holds for MSD data obtained from simulation, which instead are serially correlated and usually have unequal variances. As a consequence, the OLS is statistically inefficient, giving a relatively large statistical uncertainty in D̂*. Furthermore, the textbook OLS expression for the uncertainty in D̂* significantly underestimates the true uncertainty in this estimate.^21^ This underestimated uncertainty may give overconfidence in the accuracy of values of D* estimated using the OLS, and propagating these uncertainties in any downstream analyses may result in faulty inferences. While the uncertainty associated with OLS estimates of D* can, in principle, be accurately estimated by directly sampling over multiple repeated simulations, this approach can greatly increase the total computational cost and is often impractical.

Here we describe an approximate Bayesian regression method for estimating D* with near-maximal statistical efficiency while accurately estimating the corresponding statistical uncertainty using data from a single simulation. We model the statistical population of simulation MSDs as a multivariate normal distribution using an analytical covariance matrix derived for an equivalent system of freely diffusing particles, with this covariance matrix parametrized from the observed simulation data. We then use Markov chain Monte Carlo to sample the posterior distribution of linear models compatible with this multivariate normal model. The resulting posterior distribution provides an efficient estimate for D* and allows the associated statistical uncertainty in D̂* to be accurately quantified. This method is implemented in the open-source Python package kinisi.^37^

## II Background

For a simulation of equivalent particles, the observed mean squared displacement as a function of time, *x*(*t*), can be computed as an average over equivalent particles and time origins:
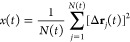
2where *N*(*t*) is the total number of observed squared displacements at time *t*. The resulting observed MSD is a vector, ***x***, with individual elements *x*_*i*_. Each element of this vector differs from the true ensemble-average MSD for that time by some unknown amount. Fitting a linear model to ***x*** gives an estimated self-diffusion coefficient, D̂*, which again differs from the true self-diffusion coefficient, D*, by some unknown amount.

Performing repeated simulations starting from different random seeds or with different histories will produce a set of replica trajectories, where each trajectory gives a different, statistically equivalent, observed MSD. The set of all possible replica trajectories defines a population of hypothetical observed MSDs, and the MSD obtained from any one trajectory can be considered a random sample, ***X***, drawn from the multivariate probability distribution that describes this population, i.e, ***X*** ∼ *p*(***x***). Each potential MSD sample could, in principle, be fitted to a linear model to obtain a corresponding estimate for the self-diffusion coefficient: ***X*** → D̂*. The population of all such estimates therefore defines a probability distribution *p*(*D̂*)*. The estimated diffusion coefficient obtained from a single simulation corresponds to a random sample drawn from this distribution, while the uncertainty in D̂* is described by the shape of the full distribution *p*(*D̂*)*.

The statistical properties of *p*(D̂*) depend on both the input MSD data and the choice of regression scheme used to obtain a “best fit” linear model. An optimal estimation scheme for D* should be unbiased, i.e., the expected value, (D̂*), should equal the true self-diffusion coefficient D*, and should be maximally statistically efficient, i.e., the spread of *p*(D̂*) around D* should be minimized. An estimation scheme should also provide an accurate estimate of the uncertainty in D̂* to allow this estimated parameter to be used in subsequent inferential analysis.

**Figure 1:**
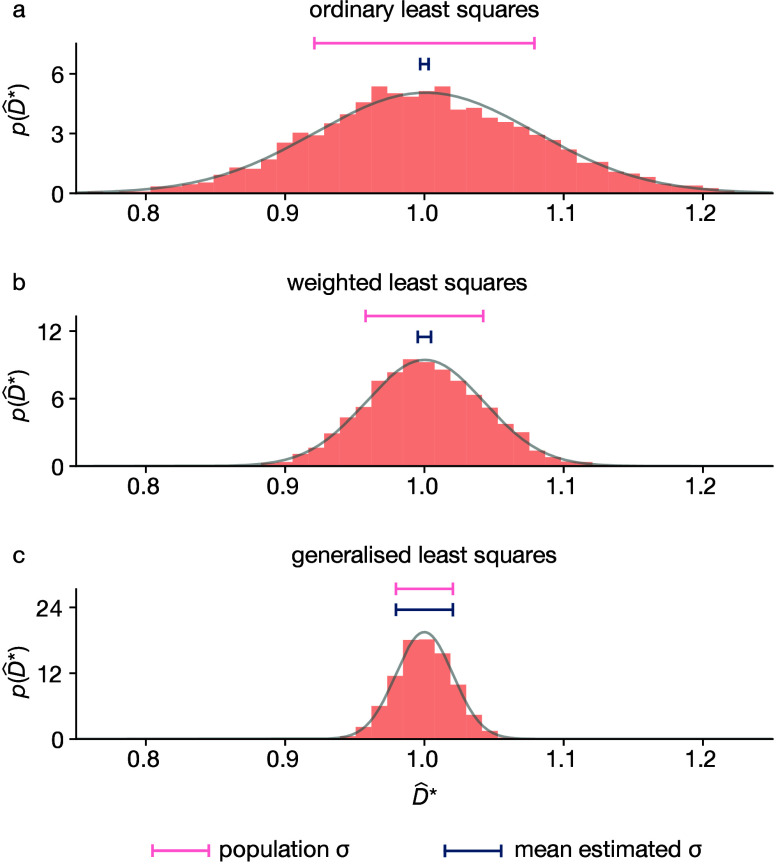
Example distributions of estimated self-diffusion coefficients, D̂*, calculated using (a) ordinary least-squares (OLS), (b) weighted least-squares (WLS), and (c) generalized least-squares (GLS), from MSD data from 4096 individual simulations of 128 particles undergoing a 128 step 3D lattice random walk with a step size chosen so that the true diffusion coefficient (D*) is equal to 1. In each panel, the gray curve shows the best-fit normal distribution for the simulation data, the upper horizontal bar shows the standard deviation of this distribution, and the lower horizontal bar shows the average estimated standard deviation given by the analytical expression for σ[*p*(D̂*)] for each regression method.

For data that are both statistically independent and identically normally distributed, ordinary least-squares regression (OLS) is unbiased and statistically efficient and gives accurate estimates of the uncertainties in the resulting regression coefficients. MSD data obtained from simulation, however, are neither statistically independent nor identically distributed. The variances, σ^2^[*x*_*i*_], are correlated since the displacement of each particle at time *t* + Δ*t* is necessarily similar to its displacement at time *t*, and hence, *x*(*t*) is similar to *x*(*t* + Δ*t*). These variances are also typically unequal—the data are heteroscedastic.^21−23^ Because the key assumptions of the OLS method are not valid for MSD data, OLS gives statistically inefficient estimates of D*, while the estimated regression uncertainties obtained from the standard OLS statistical formulas significantly underestimate the true uncertainty in *p*(*D̂*_OLS_^*^) (Figure 1a).

Some improvement can be made by using weighted least-squares (WLS) (Figure 1b), where the residual for each observed MSD value is weighted by the reciprocal of its variance, 1/(σ^2^[*x*_*i*_]). Like OLS, WLS is an unbiased estimator, and for heteroscedastic data it has higher statistical efficiency than OLS. WLS still neglects correlations in ***x***, however, and is therefore statistically inefficient, and the WLS-estimated uncertainties for the regression coefficients still underestimate the true uncertainty in *p*(*D̂̂*_WLS_^*^).

To optimally estimate the true ensemble-average MSD, and hence D*, from simulation data, it is necessary to account for both the changing variance and the correlation structure of ***x***. Within the framework of linear regression, this can be achieved using generalized least-squares (GLS). GLS gives estimated regression coefficients, β̂, via
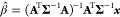
3where **A** is the model matrix [**1t**], in which ***t*** is the vector of observed times, and **Σ** is the covariance matrix for the observed MSD values. For correlated heteroscedastic data, such as MSD data, GLS offers the theoretical maximum statistical efficiency—it achieves the Cramér–Rao bound^24−28^—and provides accurate analytical estimates of the uncertainty in the predicted regression coefficients (Figure 1c).

An alternative method for estimating the ensemble-average MSD, and thus D̂*, from simulation data is Bayesian regression. Like GLS, Bayesian regression can take into account both the changing variance and the correlation structure inherent in the data. Rather than providing a singular “best-fit” estimate like GLS, Bayesian regression produces a posterior joint probability distribution for the regression coefficients. The mean of this distribution serves as a point estimate of the coefficients and, in the absence of additional prior information, is equal to the estimate obtained from GLS, while the spread of the distribution quantifies the uncertainty in these estimates. For data that are both heteroscedastic and correlated, such as MSD data from simulations, Bayesian regression, like GLS, is formally fully statistically efficient.

The estimation of D* from some observed MSD data, ***x***, using Bayesian regression proceeds by computing the posterior probability distribution *p*(***m***|***x***) for a linear model ***m*** = 6D*t + *c*, where D* and *c* are parameters to be estimated. This posterior distribution is described by Bayes’ theorem:
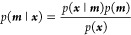
4where *p*(***x***|***m***) is the probability of observing data ***x*** given model ***m***, often described as the “likelihood”, and *p*(***x***) is the marginal probability of the observed data ***x***. Integrating over *p*(***m***|***x***) with respect to *c* yields the marginal posterior distribution *p*(D*|***x***), from which the best point estimate for D̂* and distribution variance σ̂^2^[D̂*] can be computed.

Given a sufficiently large number of observed squared displacements at each time *t*, the central limit theorem applies, and ***x*** can be considered a sample from a multivariate normal distribution with log-likelihood
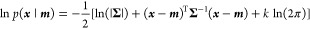
5where **Σ** is the observed MSD covariance matrix and *k* is the length of the vector ***x***, i.e., the number of time intervals for which we have observed MSD data. Provided that this likelihood function can be calculated, one can compute the posterior distribution *p*(***m***|***x***) via eq 4 to obtain an optimally efficient point estimate for D* and a complete description of the associated uncertainty in D̂*.

## III Approximating **Σ** from Simulation Data

The practical application of Bayesian regression or GLS requires the covariance matrix for the observed MSD, **Σ**, which is generally unknown. To proceed, we approximate **Σ** with a model covariance matrix, **Σ′**, with a known analytical form that we parametrize from the available simulation data. Provided that the correlation structure of **Σ′** is similar to that of **Σ**, this model correlation matrix can be used in approximate Bayesian or GLS schemes to estimate the ensemble-average MSD, and hence D*, with high efficiency and accurate estimated uncertainties.

We model the covariance matrix for the observed MSD from a given simulation by using the covariance matrix for the MSD of an equivalent system of freely diffusing particles, **Σ′**. We note that estimating D̂* by fitting a linear model to observed MSD data implicity assumes that these data sample the long-time limit where the Einstein relation (eq 1) is valid. In this long-time diffusive regime, all systems of mobile particles are statistically equivalent under rescaling by D* and hence have the same MSD covariance structure **Σ**.

For observed MSDs computed by averaging over nonoverlapping time windows, the covariance matrix **Σ′**, in the long-time limit, has elements (see the Supporting Information (SI))
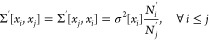
6where σ^2^[*x*_*i*_] are the time-dependent variances of the observed MSD and *N*_*i*_^′^ is the total number of nonoverlapping observed squared displacements for time interval *i*. We estimate the variances σ^2^[*x*_*i*_] using the standard result that the variance of the mean of a sample scales inversely with the number of independent constituent observations. Specifically, we compute an estimated variance σ̂^2^[*x*_*i*_] by rescaling the observed variance of the squared displacement for time interval *i* by the number of numerically independent contributing subtrajectories, *N*_*i*_*′*, which is given by the number of mutually nonoverlapping time windows of length *i* multiplied by the number of mobile particles, summed over all simulations used to compute the MSD:
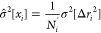
7By rescaling by the number of numerically independent contributing subtrajectories rather than by the total number of observed squared displacements for time window *i*, we account for correlations between the squared displacements of each particle computed from overlapping time windows (further details are provided in the SI).

**Figure 2:**
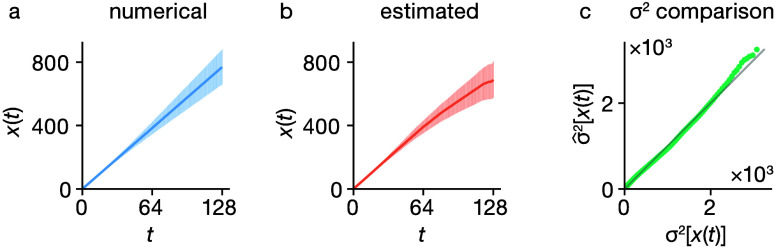
Comparison of the numerical variance in the observed MSD from multiple replica simulations and the estimated variance in the observed MSD given by rescaling the variance in observed squared displacements (eq 7). (a) Mean observed MSD from 4096 simulations of 128 particles undergoing a 3D lattice random walk of 128 steps per particle, with error bars of ±2σ[*x*_*i*_]. (b) MSD from just one simulation, with error bars of ±2σ̂[*x*_*i*_], obtained via eq 7. (c) Plot of the numerical variance against the estimated variance from a single simulation as a function of time step *t*.

The estimated variance σ̂^2^[***x***] can be calculated from a single simulation trajectory and provides an accurate estimate of the true variance σ^2^[***x***].^29−31^ To demonstrate this, we performed 4096 independent simulations of 128 particles undergoing a 3D cubic lattice random walk of 128 steps per particle. Using data from all 4096 simulations, we first computed the true simulation MSD and its variance (Figure 2a). We also computed the MSD and estimated variance using data from a single simulation trajectory (Figure 2b) using the scheme described above. A quantitative comparison between the true MSD variance and the single-trajectory estimated MSD variance is made in Figure 2c: the close numerical agreement confirms that eq 7 can be used to estimate σ^2^[***x***], which can then be used to parametrize the model covariance matrix **Σ′** via eq 6.

The practical implementation of both GLS and Bayesian regression requires that the covariance matrix **Σ′** is invertible (positive-definite) (see eqs 3 and 5). The estimated MSD variances derived from simulation data via eq 7, however, are statistically noisy, and using these to directly parametrize **Σ′** can yield matrices with high condition numbers, resulting in numerical instabilities when these are used in eq 5, or matrices that are singular and noninvertible. To make our scheme numerically tractable, we recondition the estimated covariance matrix obtained from eq 6 using the minimum eigenvalue method.^32^ This approach ensures that the condition number for the resulting covariance matrix is equal to a user-defined parameter that can be set to produce an invertible matrix that allows for numerically stable GLS or Bayesian regression.

**Figure 3:**
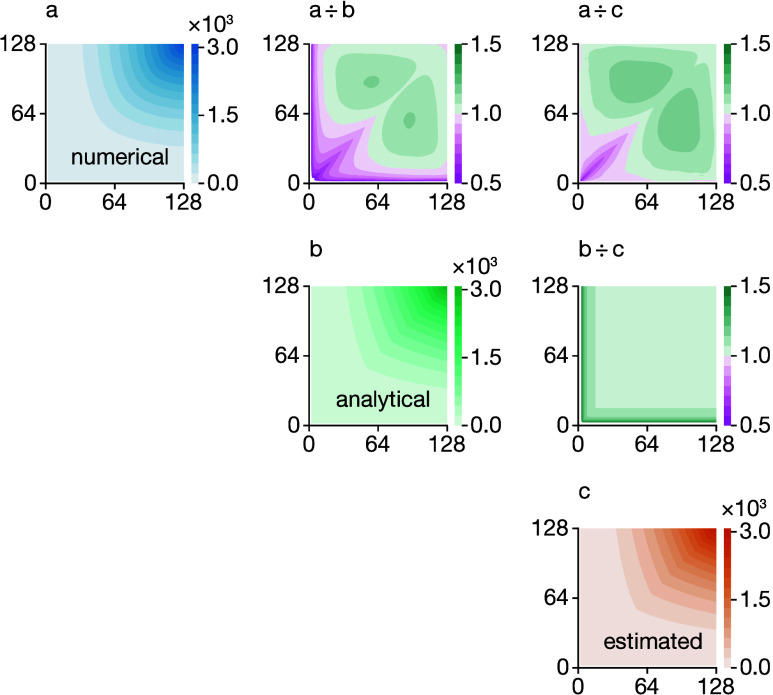
(a) Numerical MSD covariance matrix **Σ** calculated using MSD data from 4096 simulations of 128 particles undergoing a 3D lattice random walk of 128 steps per particle. (b) Analytical MSD covariance matrix **Σ′** (eq 6), parametrized using analytical long-time-limit random-walk variances σ^2^[*x*_*i*_]. (c) MSD covariance matrix obtained by applying the numerical scheme described in the main text to each individual random walk simulation, averaged over all 4096 such simulations. Color bars in (a–c) show the covariance, Σ[*x*_*i*_, *x*_*j*_]. The off-diagonal panels show difference plots computed as per-element ratios between pairs of covariance matrices (a–c).

To illustrate the accuracy of the numerical procedure for deriving the model covariance matrix, **Σ′**, we present in Figure 3 the MSD covariance matrix for 4096 random walk simulations, as described above, at three differing levels of approximation: Figure 3a shows the numerically converged covariance matrix, **Σ**, computed using the data from all 4096 simulations; Figure 3b shows the corresponding analytical covariance matrix, **Σ′**, as defined by eq 6 and parametrized using analytical long-time-limit variances σ^2^[*x*_*i*_]; and Figure 3c shows the average estimated matrix obtained by parametrizing eq 6 using variances estimated from a single simulation trajectory, then reconditioning, with the average taken over all 4096 matrices obtained from the 4096 input simulations. While the analytical and average estimated covariance matrices show some systematic deviation from the numerically converged covariance matrix, the general correlation structure is preserved. The discrepancy between the model and numerical covariance matrices largely stems from the approximation made in deriving the analytical form that *t* is large, which leads to an overestimation of the variance at low *t*. Despite this, the average estimated covariance matrix reproduces well the correlation structure of the true numerical covariance matrix, and as we show below, the covariance matrices estimated from individual simulation trajectories can be used within approximate GLS or Bayesian regression schemes to estimate D* and σ^2^[D̂*].

## IV Validation

To demonstrate the complete approximate Bayesian regression scheme, we present two example use-cases. First, we consider a simple 3D lattice random walk; in this case the true self-diffusion coefficient D* is specified by the simulation parameters and a well-converged numerical covariance matrix can be obtained at relatively low computational cost, allowing us to directly compare the estimates produced by our method to “best case” estimates from a hypothetical method with access to the true covariance matrix. Second, we consider an example real-world system, the lithium-ion solid electrolyte Li_7_La_3_Zr_2_O_12_ (LLZO), which represents an application of our method to a well-studied material of practical interest for solid-state lithium-ion batteries.^33−36^

**Figure 4:**
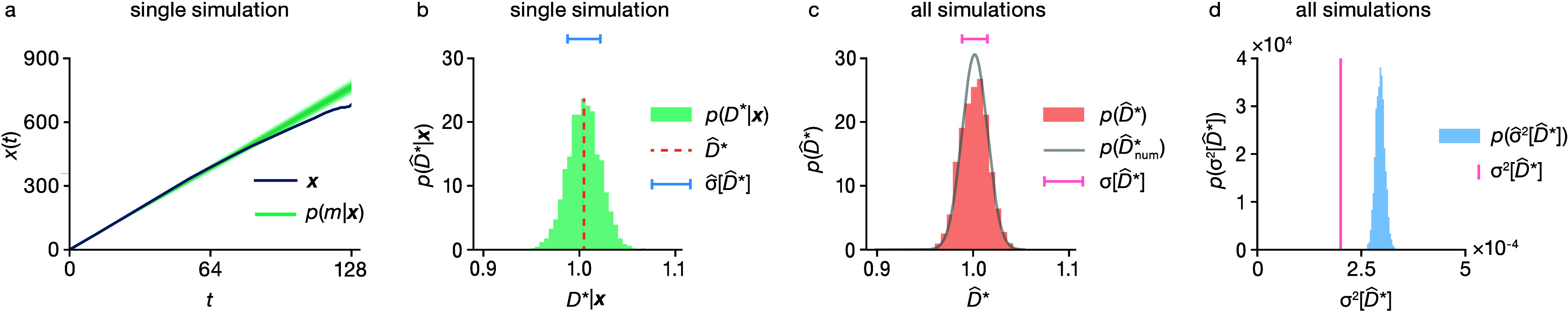
(a) Observed MSD from a single simulation of 128 particles undergoing a 3D lattice random walk of 128 steps per particle (dark line). The green shading shows the corresponding posterior distribution *p*(***m***|***x***) of linear models compatible with the observed MSD data ***x***, calculated by using the scheme described in the main text. The variegated shading indicates compatibility intervals of (1, 2, and 3) σ[*p*(***m***|***x***)]. (b) Marginal posterior distribution *p*(D̂*|***x***) obtained from the posterior distribution of linear models in (a). The mean of this distribution gives the point estimate D̂* for these simulation input data. The blue horizontal bar shows an interval of 1 standard deviation in *p*(D̂*|***x***). (c) Probability distribution of point estimates *p*(D̂*) obtained from 4096 random walk simulations. Each simulation was analyzed as in (a) and (b) to yield a single corresponding point estimate D̂*. The gray line shows the distribution of point estimates, *p*(*D̂*_num_^*^), obtained using Bayesian regression with a mean vector and numerical covariance matrix derived from the complete dataset of all 4096 simulations. The pink horizontal bar shows an interval of 1 standard deviation in *p*(D̂*). (d) Probability distribution of estimated variances, σ̂^2^[D̂*], for individual random walk simulations, compared to the true sample variance (pink vertical line) σ^2^[D̂*].

Figure 4a shows the observed MSD from a single 3D lattice random walk simulation, along with the estimated posterior distribution of linear models compatible with the observed MSD data, *p*(***m***|***x***), calculated via eqs 4 and 5. The corresponding marginal posterior distribution of estimated diffusion coefficients *p*(D*|***x***) is shown in Figure 4b; this distribution is approximately Gaussian and is centered close to the true self-diffusion coefficient D* = 1, demonstrating that for this example trajectory we obtain a good point estimate of D*.

To evaluate the overall performance of our method, we repeat our analysis on the full set of 4096 random walk simulations. Figure 4c presents a histogram of the resulting point estimates of D*, with each estimate derived as the mean of the posterior distribution *p*(D*|***x***) using input data from each individual simulation. We also show the probability distribution of estimated diffusion coefficients obtained using Bayesian regression with a mean vector and covariance matrix derived numerically from all 4096 simulations (solid line). This latter distribution represents the distribution of “best possible” estimates of D* and exhibits the minimum theoretical variance. The close agreement between these two distributions demonstrates that our approximate Bayesian regression scheme yields nearly optimal estimates of D* using data from individual simulations. The distribution of estimated diffusion coefficients from single simulations is slightly broader than the numerical results. This minor deviation is a consequence of the noise present in the data obtained from a single simulation trajectory.

We next consider the degree to which our method can quantify the uncertainty in D̂* when using input data from a single simulation. Figure 4d shows the distribution of estimated variances σ̂^2^[D̂*], with each sample calculated from an individual simulation trajectory. We also show the true variance of individual point estimates, σ^2^[D̂*], which characterizes the spread of the histogram in Figure 4c. The distribution of estimated variances is biased relative to the true variance due to numerical differences between the true covariance matrix **Σ** and the estimated covariance matrix **Σ′** (further details are provided in the SI). In general, however, the distribution of the estimated variance shows good agreement with the true sample variance. Notably, the precision of this estimate is significantly greater than that obtained using OLS or WLS and their respective textbook statistical formulas.

We also benchmark our method using data from simulations of the lithium-ion solid electrolyte cubic Li_7_La_3_Zr_2_O_12_ (c-LLZO). We performed a single simulation of 1536 atoms (448 Li ions) at 700 K for 6 ns (full simulation details are given in Methods). To generate multiple statistically equivalent trajectories, we partitioned the output simulation data into 192 effective trajectories, each ∼500 ps in length and containing data for 28 lithium ions, which were selected randomly from the complete set of 448 lithium ions without replacement. We then performed approximate Bayesian regression, as above, on each effective trajectory, excluding the first 10 ps of MSD data in each case to remove short-time data corresponding to the ballistic and subdiffusive regimes.^23,34^

**Figure 5:**
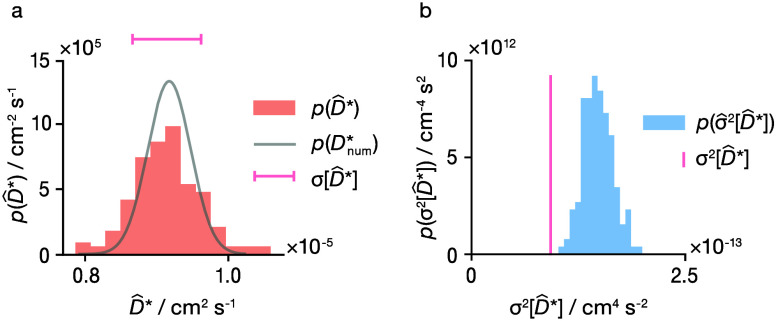
(a) Probability distribution of point estimates *p*(D̂*) for 192 effective simulations of LLZO (orange histogram). The gray line shows the distribution *p*(*D̂̂*_num_^*^) obtained using Bayesian regression with the complete LLZO dataset as input. The pink bar shows an interval of 1 standard deviation σ[*p*(D̂*)]. (b) Probability distribution of estimated variances, σ̂^2^[D̂*], for individual LLZO effective simulations compared to the true sample variance σ^2^[D̂*] (pink vertical line).

The resulting distribution of the point estimates, D̂*, from analysis of all 192 effective trajectories is shown in Figure 5a. As above, we also show the corresponding distribution of D̂* estimates obtained via Bayesian regression using a well-converged numerical covariance matrix calculated from the full LLZO dataset. The distribution *p*(D̂*) obtained using the model covariance matrix and parametrized separately for each individual effective simulation is highly similar to that obtained using the aggregate numerical covariance matrix calculated from the complete simulation dataset. This close agreement mirrors the results for our random walk simulations and confirms that our method yields accurate and statistically efficient estimates for D*, even for real-world simulation data.

We also consider the probability distribution of estimates of the variance in D̂* calculated for each effective trajectory (Figure 5b), which we compare to the true variance in D̂* for our method, i.e., the variance of the histogram in Figure 5a. While the estimated variances deviate somewhat from the true distribution *p*(σ^2^[D̂*]), the agreement is reasonable and mirrors our results for the random walk simulations. Hence, our method provides reasonably accurate estimates of the uncertainty in D̂* for our c-LLZO dataset, even when applied to single effective trajectories with limited displacement data (only 28 mobile ions and 500 ps simulation length).

## V σ^2^[D̂*] Scaling and Comparison to OLS, WLS, and GLS

**Figure 6:**
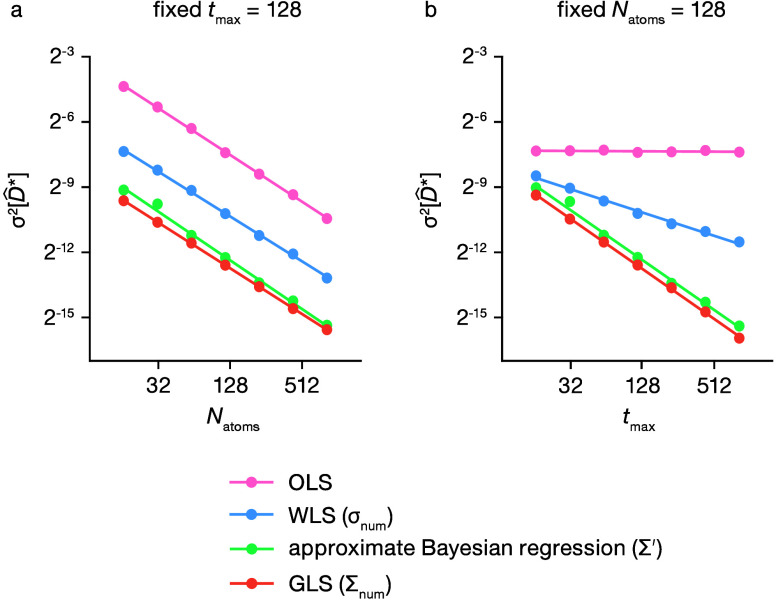
Scaling of σ^2^[D̂*] with the simulation size for the OLS (pink), WLS (blue), our approximate Bayesian regression method (green), and GLS (orange). (a) Scaling versus number of mobile particles, *N*_atoms_. (b) Scaling versus total simulation time, *t*_max_. Solid lines show fitted power law relationships for each dataset. The WLS and GLS data are obtained using numerically determined variances and covariance, respectively, from a set of 512 repeat simulations for each combination of *N*_atoms_ and *t*_max_.

Figure 6 presents an analysis of the variation in σ^2^[D̂*] as the number of mobile particles (Figure 6a) and the total simulation time (number of steps) (Figure 6b) are changed. We compare four methods for estimating D* from the observed MSD data: OLS, WLS, the approximate Bayesian regression method described here, and GLS. When estimating D* using WLS and GLS, we calculate the variances and the covariance matrix, respectively, numerically, using the complete set of 512 simulations. Each data point in Figure 6 represents the variance across point estimates of D* derived from 512 individual 3D lattice random walk simulations for each combination of *N*_atoms_ and *t*_max_. The GLS dataset corresponds to an optimally efficient estimator for linear regression of observed MSD data and is equivalent to performing Bayesian regression with the converged numerical covariance matrix and an uninformative prior.

Our approximate Bayesian regression method performs similarly to GLS with a numerically converged covariance matrix and gives significantly reduced uncertainty in D̂* compared to that of OLS or WLS for all simulation sizes and lengths considered. Moreover, our method scales better than OLS or WLS as the total simulation time is increased. This approximate Bayesian regression method therefore presents a significant improvement over more conventional methods, such as OLS and WLS, by enabling more precise estimates of D* across varied simulation sizes at equivalent computational cost.

## VI Summary and Discussion

We have introduced and demonstrated an approximate Bayesian regression method for estimating the self-diffusion coefficient, D*, from molecular dynamics simulation data. We consider the observed mean squared displacement data from a single simulation as a random sample, ***X***, from a population of potential MSDs generated by equivalent replica simulations, ***X*** ∼ *p*(***x***). We model this population using a multivariate normal distribution, , with mean vector ***m*** = 6D*t + *c*, where D* and *c* are model parameters to be determined.

To model the covariance matrix, we used an analytical solution derived for an equivalent system of freely diffusing particles. To parametrize this model covariance matrix, we renormalized the variance of the observed squared displacements from the input simulation trajectory, followed by a reconditioning step to ensure a positive-definite matrix. The resulting model covariance matrix preserves the correlation structure of the true simulation MSD covariance matrix and gives a multivariate normal model for the population of observable simulation MSDs that depends solely on the model parameters D* and *c*.

We used Markov chain Monte Carlo to sample the posterior distribution of linear models compatible with the observed MSD data. This approach yields a marginal posterior distribution, *p*(D*|***x***), that gives a statistically efficient point estimate for D* and allows the associated statistical uncertainty, σ^2^[D̂*], to be quantified.

We have benchmarked our approach using simulation data for an ideal 3D lattice random walk and for the lithium-ion solid electrolyte Li_7_La_3_Zr_2_O_12_ (LLZO). In both cases, we obtain a distribution of estimates for D* that closely matches the theoretically optimal distribution obtained using a well-converged numerical covariance matrix derived from a large number of replica simulation trajectories.

We obtain estimates for D* that are unbiased, with near-optimal statistical efficiency, using input data from single simulation trajectories. The approximate Bayesian regression scheme therefore provides more accurate single-point estimates of the self-diffusion coefficient than the commonly used methods of OLS or WLS when applied to the same input simulation data. The improved statistical efficiency of this method compared to OLS or WLS enables the estimation of D* with equivalent accuracy from considerably smaller simulations—either in terms of time scale or system size. This reduces the overall computational cost compared to studies that use OLS or WLS for estimating a linear fit to simulation MSD data. Alternatively, this approach enables the estimation of D* with greater precision given simulation trajectories of equal size.

Our method also provides reasonable estimates of the statistical uncertainty in the estimated value D̂*, in contrast to OLS and WLS, which systematically significantly underestimate the uncertainty in regression coefficients when applied to simulated MSD data. While these estimated statistical uncertainties can still differ from the true (but unknown) uncertainty in D̂*, particularly when using short-time-scale simulation data, they allow for scientifically meaningful comparisons to be made between estimated diffusion coefficients across different systems or under varying conditions, such as changes in temperature, or between computational findings and experimental results. Furthermore, these uncertainties allow for quantitative downstream analysis, such as the application of Arrhenius- or non-Arrhenius-type models to describe the temperature dependence of self-diffusion.

The approximate Bayesian regression scheme presented here provides a statistically efficient means of estimating the self-diffusion coefficient, D*, from molecular dynamics simulation data. It improves upon textbook approaches by providing accurate point estimates of D* with near-optimal statistical efficiency while also providing a reasonable description of the uncertainty in these estimates. The high statistical efficiency of our method allows for the use of smaller simulations, which can significantly reduce computational costs. Overall, our method offers significant advantages over more conventional methods of estimating self-diffusion coefficients from atomistic simulations. We have implemented this procedure in the open-source package kinisi,^37^ which we hope will support its use within the broader simulation community.

## VII Methods

### A Numerical Implementation in KINISI

kinisi-1.1.0 was used for all analyses presented in this work.

When calculating the observed mean squared displacement at each time interval *t* (see eq 2), kinisi uses overlapping sliding window sampling. For a given time interval, *t*, the maximum number of observations is *N*_atoms_ × (*N*_*t*_ – *i*) displacements, where *N*_atoms_ is the number of mobile atoms, *N*_*t*_ is the total number of time steps, and *i* is the index of the time step (where 1 is the index for the shortest time step). To estimate the variance of the observed MSD, we rescale the variance of observed squared displacements by the number of numerically independent subtrajectories in the simulation, *N*_*i*_^′^ = *N*_atoms_ × *N*_*t*_/*i*, as presented in eq 7.

The parametrization of the covariance matrix from the variances σ^2^[*x*_*i*_] and the number of independent observations *N*_*i*_^′^ is defined by eq 6. The covariance matrix is constructed only for values of *t* where the particle motion is considered to be in the long-time diffusive limit. In practice, this threshold is set by the user to a value appropriate for their system and simulation data. For the examples presented in the main paper, we consider particles to be in the diffusive regime from *t* = 2 for the random walk trajectories and from *t* = 10 ps for the LLZO simulations. The covariance matrix is reconditioned using the minimum eigenvalue method^32^ with a maximum condition number of 1 × 10^16^ for all simulations.

To estimate D̂* from a given set of MSD data, kinisi uses ordinary least-squares to obtain an initial guess for the gradient and intercept of the linear model that best describes the observed MSD. This initial guess is then used as the starting point for minimizing the negative maximum a posteriori (the peak of the posterior distribution as per eq 4) with the improper prior that D* ≥ 0.^38−41^ We note that the Bayesian regression formalism presented here allows for the use of alternative informative priors in cases where the user has some prior knowledge of the system being simulated that they wish to incorporate into their analysis. The log-likelihood calculation (eq 5) uses the Moore–Penrose generalization of the inverse of a Hermitian matrix.^42−44^

To sample the joint posterior probability distribution of the linear model, kinisi uses the emcee package,^45^ which implements Goodman and Weare’s affine invariant Markov chain Monte Carlo ensemble sampler.^46^ When sampling *p*(D*|***m***), we again apply the improper prior D* ≥ 0. The sampling process uses 32 walkers for 1500 steps, with the first 500 steps discarded as a burn-in period. The sampled chains are thinned such that only every 10th value is retained, yielding 3200 points sampled from the posterior distribution *p*(D*|***m***). These points can then be plotted as a histogram (as in Figure 4b), and summary statistics D̂* and σ̂^2^[D̂*] can be derived.

### B LLZO Simulations

Classical molecular dynamics simulations were run using the metalwalls code.^47^ We used the DIPPIM polarizable ion force field as parametrized by Burbano et al.^34^ We simulated the cubic phase of LLZO in the *NVT* ensemble at a temperature of 700 K. Simulations were run for 6 ns with a 0.5 fs time step. To control the temperature, we used a Nosé–Hoover thermostat with a relaxation time of 121 fs (5000 *ℏ*/*E*_h_).^48−50^ Simulations were performed using 2 × 2 × 2 supercells with 1536 atoms following the same protocol as in ref (34).

## Data Availability

Additional supporting information is available at ref (51) under an MIT license, including a complete set of analysis/plotting scripts allowing for a fully reproducible and automated analysis workflow, using showyourwork.^52^ The LLZO raw simulation trajectories are available on Zenodo shared under a CC BY-SA 4.0 license.^53^ The method outlined in this work is implemented in the open-source Python package kinisi,^37^ which is available under an MIT license.

## Supporting Information Available

Derivation of long-time-limit covariance matrix for a system of freely diffusing particles, comparison of variance rescaling (eq 7) and block renormalization approaches, discussion of bias in the distribution of estimated variance of the estimated diffusion coefficient, and a comparison of OLS, WLS, and GLS as estimators for *D** applied to MSD data from simulations of LLZO (PDF)
