# Supplementary material for: Accurate Estimation of Diffusion Coefficients and their Uncertainties from Computer Simulation
Source: J Chem Theory Comput. 2024 Dec 30;21(1):79–87. doi: 10.1021/acs.jctc.4c01249 (PMC11736684; doi:10.1021/acs.jctc.4c01249)
Supplement: Supplementary file 1 — ct4c01249_si_001.pdf [file ct4c01249_si_001.pdf]

Supplemental Material for "Accurate Estimation of Diffusion Coefficients and their Uncertainties from Computer Simulation"

Andrew R. McCluskey,[1,2,3,*] Samuel W. Coles,[4,5] and Benjamin J. Morgan[4,5,†]
[1] *Centre for Computational Chemistry, School of Chemistry, University of Bristol, Cantock's Close, Bristol, BS8 1TS, UK.*
[2] *European Spallation Source ERIC, Data Management and Software Centre, Asmussens Allé 305, DK-2800 Kongens Lyngby, DK.*
[3] *Diamond Light Source, Harwell Campus, Didcot, OX11 0DE, UK.*
[4] *Department of Chemistry, University of Bath, Claverton Down, Bath, BA2 7AY, UK*
[5] *The Faraday Institution, Quad One, Harwell Science and Innovation Campus, Didcot, OX11 0RA, UK*

This document presents supplementary material for the manuscript "Accurate Estimation of Diffusion Coefficients and their Uncertainties from Computer Simulation". It contains the following sections:

S-I. The derivation of the covariance matrix in the long-time limit for freely diffusion particles.

S-II. Further details of the variance rescaling method for estimating $\sigma^2[x_i]$, described in the main text, and a comparison to the block renormalisation method for estimating the variance of the mean for serially-correlated data of Flyvbjerg and Petersen [1].

S-III. Discussion of the origin of bias in the distribution of the estimated variance of the estimated diffusion coefficient, $p(\widehat{\sigma}^2[\widehat{D}^*])$.

S-IV. A comparison of OLS, WLS, and GLS as estimators for D^* applied to MSD data from simulations of $Li_7La_3Zr_2O_{12}$ (LLZO).

A repository containing the analysis and plotting code used to generate all results and figures in the main manuscript and this supplemental material document is available at `www.github.com/arm61/msd-errors` [2], under MIT (code) and CC BY-SA 4.0 (figures and text) licenses. This repository includes a fully reproducible showyourwork workflow, which allows complete reproduction of the analysis, plotting of figures and compilation of the manuscripts. The corresponding LLZO simulation datasets are openly available under the CC BY-SA 4.0 licence [3].

S-I: Derivation of the long-time limit covariance matrix for a system of freely diffusing particles.

In the main manuscript we present the result that the covariance matrix for a system of freely diffusing particles, in the long-time limit, has the form

$$\Sigma'\left[x_i, x_j\right] = \Sigma'\left[x_j, x_i\right] = \sigma^2[x_i]\frac{N_i'}{N_j'}, \quad \forall\, i \leq j, \tag{S-1}$$

where x_i is the observed mean-squared displacement (MSD) for time interval i and N_i' is the number of statistically independent observed squared displacements averaged over to compute the mean value.

To derive this result, we first present a derivation of the expected variance for the MSD at timestep i, $\sigma^2[\boldsymbol{x}]$, following the approach of Smith and Gillan [4]. We then derive an expression for the covariance $\Sigma'\left[x_i, x_j\right]$ to obtain the result above.

For a single particle undergoing a one-dimensional random walk with step size κ, each step gives a displacement $h = \pm\kappa$. After n steps, the MSD, x_n, is given by

$$\begin{aligned} x_n &= \left[\sum_i^n h_i\right]^2 \\ &= \sum_i^n \sum_j^n h_i h_j \\ &= \sum_i^n h_i^2 + \sum_i^n \sum_{j\neq i}^n h_i h_j. \end{aligned} \tag{S-2}$$

The expected MSD in the long-time limit, $\mathbb{E}(x_n) = \langle x_n \rangle$, is obtained by averaging over all permutations of h_i and h_j:

$$\langle x_n \rangle = \sum_i^n \left\langle h_i^2 \right\rangle + \sum_i^n \sum_{j\neq i}^n \left\langle h_i h_j \right\rangle. \tag{S-3}$$

For a random walk, the second term averages to zero for all h_i and h_j, and

$$\begin{aligned} \langle x_n \rangle &= \sum_i^n \left\langle h_i^2 \right\rangle \\ &= n\kappa^2. \end{aligned} \tag{S-4}$$

Hence the expected value for the mean-squared displacement increases linearly with the number of steps taken.

* andrew.mccluskey@bristol.ac.uk
† b.j.morgan@bath.ac.uk

The variance in the observed MSD, $\sigma^2[x_n]$, is given by the standard statistical formula

$$\sigma^2[x_n] = \left\langle [x_n - \langle x_n \rangle]^2 \right\rangle, \tag{S-5}$$

which can be expanded as

$$\begin{aligned} \sigma^2[x_n] &= \langle x_n^2 \rangle - 2 \langle x_n \rangle \langle x_n \rangle + \langle x_n \rangle^2, \\ &= \langle x_n^2 \rangle - \langle x_n \rangle^2. \end{aligned} \tag{S-6}$$

The first term can be expanded in terms of displacements h as

$$\langle x_n^2 \rangle = \left\langle \sum_i^n \sum_j^n \sum_k^n \sum_l^n h_i h_j h_k h_l \right\rangle, \tag{S-7}$$

which can be simplified by noting that h_i, h_j, h_k, and h_l are uncorrelated when $i \neq j \neq k \neq l$, and the only terms that contribute to the average are those where $h_i h_j h_k h_l$ is guaranteed to be non-zero:

(a) $i = j = k = l$;

(b) $(i = j) \neq (k = l)$;

(c) $(i = k) \neq (j = l)$;

(d) $(i = l) \neq (j = k)$.

From (a) we obtain

$$\left\langle \sum_i^n h_i^4 \right\rangle = n\kappa^4, \tag{S-8}$$

and from (b), (c), and (d), which are equivalent, we obtain

$$\left\langle \sum_i^n \sum_j^n h_i^2 h_j^2 \right\rangle = (n\kappa^2)^2 = n^2\kappa^4. \tag{S-9}$$

This gives

$$\langle x_n^2 \rangle = (3n^2 + n)\kappa^4, \tag{S-10}$$

which, in the limit $n \to \infty$, approaches

$$\langle x_n^2 \rangle = 3n^2\kappa^4. \tag{S-11}$$

Combining this result with Eqn. S-4, we can express the variance in the mean-squared displacement as

$$\sigma^2[x_n] = 3n^2\kappa^4 - n^2\kappa^4 = 2n^2\kappa^4, \tag{S-12}$$

i.e., $\sigma^2[x_n]$ increases quadratically with the number of steps taken, or, equivalently, with time.

Equation S-12 gives the variance of the mean squared displacement for a single particle considering a single time-origin. We can obtain improved statistics by averaging over statistically equivalent observed squared displacements (see Eqn. 2 in the main text), which can be achieved by averaging over mobile particles or by averaging over time origins. This averaging over equivalent observations reduces the variance in the observed MSD to

$$\sigma^2[x_n] = \frac{2n^2\kappa^4}{N_n'}, \tag{S-13}$$

where N_n' is the total number of statistically independent squared displacements that contribute to x_i. In the long-time limit, N_n' is given by the product of the number of mobile particles and the number of non-overlapping time-windows of length i in our simulation trajectory. Note that N_n' considers non-overlapping time windows, since mutually overlapping time-windows give correlated squared displacements.

The results for a one-dimensional lattice above (Eqns. S-4 and S-13) can be extended to a d-dimensional lattice, to give

$$\langle x_n \rangle_d = \sum^d \frac{n\kappa^2}{d} = n\kappa^2, \tag{S-14}$$

with variance

$$\sigma^2[x_n]_d = \sum^d \frac{2n^2\kappa^4}{d^2 N_n'} = \frac{2n^2\kappa^4}{dN_n'}, \tag{S-15}$$

Because each step is equally likely to move a particle along each of the d dimensions, the term n in Eqns. S-4 and S-13 is replaced here with n/d.

The analysis above can be extended to consider the covariance between two different numbers of steps, n and $n+m$, in the random walk where the expected MSDs will be

$$\begin{aligned} \langle x_n \rangle &= n\kappa^2; \\ \langle x_{n+m} \rangle &= (n+m)\kappa^2. \end{aligned} \tag{S-16}$$

The covariance between these is defined as

$$\Sigma\left[x_n, x_{n+m}\right] = \langle [x_n - \langle x_n \rangle] \left[x_{n+m} - \langle x_{n+m} \rangle\right] \rangle, \tag{S-17}$$

which can be expanded as

$$\begin{aligned} \Sigma\left[x_n, x_{n+m}\right] = \big\langle & x_n x_{n+m} - x_n \langle x_{n+m} \rangle \\ & - \langle x_n \rangle x_{n+m} + \langle x_n \rangle \langle x_{n+m} \rangle \big\rangle, \end{aligned} \tag{S-18}$$

and then reformulated to give

$$\Sigma\left[x_n, x_{n+m}\right] = \langle x_n x_{n+m} \rangle - \langle x_n \rangle \langle x_{n+m} \rangle, \tag{S-19}$$

where

$$\begin{aligned} \langle x_n \rangle \langle x_{n+m} \rangle &= x_n x_{n+m} \\ &= n\kappa^2 (n+m)\kappa^2 \\ &= n(n+m)\kappa^4 \end{aligned} \tag{S-20}$$

and, by analogy to Eqn. S-7,

$$\langle x_n x_{n+m} \rangle = \left\langle \sum_i^n \sum_j^n \sum_k^{n+m} \sum_l^{n+m} h_i h_j h_k h_l \right\rangle, \tag{S-21}$$

which we can rewrite as

$$\langle x_n x_{n+m}\rangle = \Bigg\langle \sum_{i=1}^{n}\sum_{j=1}^{n}\sum_{k=1}^{n}\sum_{l=1}^{n} h_i h_j h_k h_l + \sum_{i=1}^{n}\sum_{j=1}^{n}\sum_{k=1}^{n}\sum_{l=n+1}^{n+m} h_i h_j h_k h_l + \sum_{i=1}^{n}\sum_{j=1}^{n}\sum_{k=n+1}^{n+m}\sum_{l=1}^{n} h_i h_j h_k h_l + \sum_{i=1}^{n}\sum_{j=1}^{n}\sum_{k=n+1}^{n+m}\sum_{l=n+1}^{n+m} h_i h_j h_k h_l \Bigg\rangle. \tag{S-22}$$

The second and third terms in Eqn. S-22 tend to zero as there is an equal probability of positive and negative displacements. This reduces Eqn. S-22 to

$$\langle x_n x_{n+m}\rangle = \Bigg\langle \sum_{i=1}^{n}\sum_{j=1}^{n}\sum_{k=1}^{n}\sum_{l=1}^{n} h_i h_j h_k h_l \Bigg\rangle + \Bigg\langle \sum_{i=1}^{n}\sum_{j=1}^{n}\sum_{k=n+1}^{n+m}\sum_{l=n+1}^{n+m} h_i h_j h_k h_l \Bigg\rangle, \tag{S-23}$$

and using Eqn. S-11 gives

$$\langle x_n x_{n+m}\rangle = 3n^2\kappa^4 + \Bigg\langle \sum_{i=1}^{n}\sum_{j=1}^{n}\sum_{k=n+1}^{n+m}\sum_{l=n+1}^{n+m} h_i h_j h_k h_l \Bigg\rangle. \tag{S-24}$$

We can rewrite this as

$$\langle x_n x_{n+m}\rangle = 3n^2\kappa^4 + \Bigg\langle \sum_{i=1}^{n}\sum_{j=1}^{n} h_i h_j \Bigg\rangle \Bigg\langle \sum_{k=n+1}^{n+m}\sum_{l=n+1}^{n+m} h_k h_l \Bigg\rangle, \tag{S-25}$$

where the following holds,

$$\begin{aligned} \langle x_n x_{n+m}\rangle &= 3n^2\kappa^4 + n\kappa^2 m\kappa^2 \\ &= 3n\kappa^4 + nm\kappa^4. \end{aligned} \tag{S-26}$$

Putting this result into Eqn. S-19 allows the covariance to be written as

$$\begin{aligned} \Sigma'\left[x_n, x_{n+m}\right] &= 3n^2\kappa^4 + nm\kappa^4 - n(n+m)\kappa^4 \\ &= 3n^2\kappa^4 - n^2\kappa^4 = 2n^2\kappa^4, \end{aligned} \tag{S-27}$$

where we use the Σ' notation to identify that this is in the long-time limit.

In this case, the covariance depends only on the number of overlapping points, n, between the two time intervals. We can rationalise this by noting that for a random walk any non-overlapping points will be completely uncorrelated and therefore have a covariance of 0. Similar to the case for the variance, the covariance derived in Eqn. S-27 is that for a single particle at a single time origin. The number of independent observed squared displacements for a given covariance should be the minimum number of shared independent observed squared displacements between the two time intervals, which is N'_{n+m}. Therefore, the covariance, scaled by the number of contributing independent observations, in the long-time limit, is

$$\Sigma'\left[x_n, x_{n+m}\right] = \frac{2n^2\kappa^4}{N'_{n+m}}. \tag{S-28}$$

Similar to the MSD and the variance, the covariance can be written for d-dimensions as

$$\Sigma'\left[x_n, x_{n+m}\right] = \frac{2n^2\kappa^4}{dN'_{n+m}}. \tag{S-29}$$

The covariance can be calculated directly from the variance by recognising that both depend on the number of overlapping points, n, as follows

$$\Sigma'\left[x_n, x_{n+m}\right] = \sigma^2[x_n]\frac{N'_n}{N'_{n+m}}. \tag{S-30}$$

This is then rewritten in terms of i and j to give, Eqn. S-1.

Using the equivalence of $2dD^*t \equiv n\kappa^2$ [5], Eqns. S-1 and S-4 can be rewritten in terms of t (or t_1 and t_2) and the diffusion coefficient, for any dimensionality of lattice random walk,

$$x(t) = 2dD^*t, \tag{S-31}$$

and

$$\Sigma'\left[x(t_1), x(t_2)\right] = 8d(D^*)^2 t_1^2 \frac{N'(t_2)}{N'(t_1)}, \quad \forall\, t_1 \leq t_2. \tag{S-32}$$

S-II: Estimating $\sigma^2[x_i]$: variance rescaling versus block renormalisation

The approximate Bayesian regression scheme described in our main manuscript uses a model covariance matrix parametrised by the variance of the observed MSD as a function of time, denoted $\sigma^2[x_i]$. Generally, $\sigma^2[x_i]$ is unknown and must be estimated from the input simulation data.

In the main manuscript, we describe an estimation approach that involves rescaling the observed variance of the squared displacement for time interval i by the number of numerically-independent contributing sub-trajectories, N'_i (Eqn. 7). We define a sub-trajectory as the sequence of displacements of one particle over a time interval of length i, and consider sub-trajectories to be numerically independent under two conditions: either when they describe displacements of different particles, or when they describe displacements of the same particle but are calculated from non-overlapping time windows (see Fig. S-1).

An alternative method for estimating the variance of the mean for time-correlated data is block averaging [7],

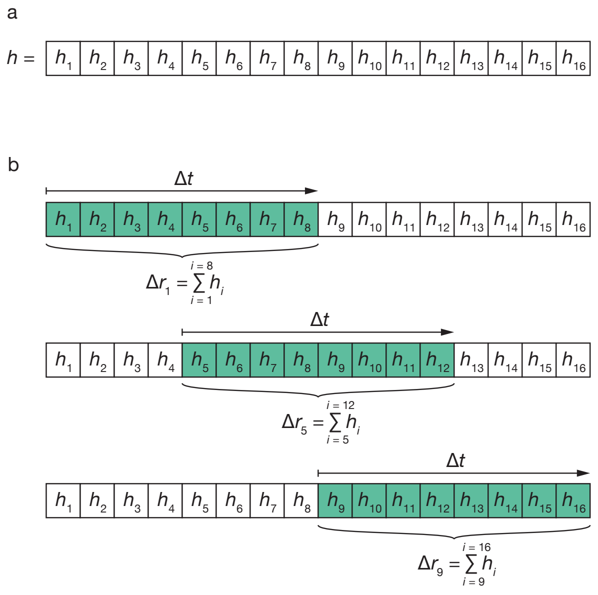

FIG. S-1. Schematic showing the construction of three statistically-equivalent sub-trajectories of length $\Delta t = 8$ from a full simulation trajectory. (a) The full trajectory consists of a series of displacements, h_i, from each simulation timestep. (b) Three exemplar sub-trajectories of length $\Delta t = 8$. Even in the case of a random walk (i.e., $h = \pm\kappa$), Δr_1 and Δr_5 are necessarily correlated, because both summations include $[h_5 + h_6 + h_7 + h_8]$. Δr_5 and Δr_9 are also obtained from mutually overlapping time windows and are therefore also correlated. For a random walk, Δr_1 and Δr_9 are uncorrelated. For a non-random (correlated) walk, non-overlapping time windows are uncorrelated in the limit that the window length, Δt, is much greater than the correlation time in h, which is the case if we are in the long-time linear region of the MSD where Eqn. 1 is valid.[a]

[a] The result that displacements calculated from overlapping time windows are correlated has been reported as an empirical result for simulations of a Lennard–Jones fluid in Ref. 6.

where the input data is divided into non-overlapping sequential “blocks”, and the set of averages calculated over each block are used for statistical analysis. A popular form of block averaging is the block renormalisation method of Flyvbjerg and Peterson [1].

The Flyvbjerg–Peterson method starts with some input data, A. If the elements of A are uncorrelated, the variance of the mean can be estimated by rescaling the variance of A:

$$\widehat{\sigma}^2[\,\overline{A}\,] = \frac{\sigma^2[A]}{L_A - 1}, \tag{S-33}$$

where L_A is the number of elements in A. If elements of A are serially correlated, however, this estimator systematically underestimates the true variance of the mean of A, and, instead, providing only an approximate lower bound:

$$\widehat{\sigma}^2[\,\overline{A}\,] \geq \frac{\sigma^2[A]}{L_A - 1}. \tag{S-34}$$

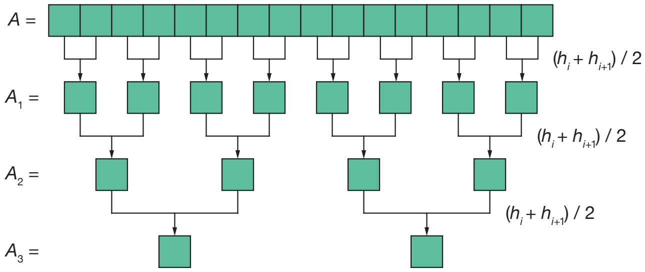

FIG. S-2. Schematic illustrating the application of repeated blocking operations with block-length 2 to generate a series of representations of the input data, A, A_1, …, A_n, as used in the block renormalisation scheme of Flyvbjerg and Peterson [1].

The method proceeds by iteratively applying “blocking” operations. The original dataset, A, is mapped to a new dataset A_1, by averaging over adjacent non-overlapping pairs of data in A (see Fig. S-2). The new dataset is half the length of the original: $L_{A_1} = \frac{1}{2}L_A$. A and A_1 have the same mean. However, we can now rescale the variance of A_1 to obtain a tighter estimated lower bound for $\sigma^2[\,\overline{A}\,]$:

$$\sigma^2[\,\overline{A}\,] \geq \frac{\sigma^2[A_1]}{L_{A_1} - 1}. \tag{S-35}$$

Under repeated blocking operations with a sufficiently large input dataset, $\sigma^2[A_n]/(L_{A_n} - 1)$ tends to $\sigma^2[\,\overline{A}\,]$. In practice, sequential blocking steps are applied to the original dataset until $\sigma^2[A_n]/(L_{A_n} - 1)$ reaches a plateau, and this plateau value is taken as the estimate for $\sigma^2[\,\overline{A}\,]$.

The Flyvbjerg–Peterson block renormalisation method is particularly useful for estimating the variance of the mean in sequential correlated data where the correlation length is unknown; for example, when estimating thermodynamic averages from simulation trajectories.

Figure S-3 shows a comparison of the variance rescaling method described in the main text with the Flyvbjerg–Petersen block renormalisation method when using both methods to estimate the $\sigma^2[x_i]$ from simulation. We applied both methods to a simulation of 128 particles undergoing a three-dimensional cubic-lattice random walk of 128 steps per particle.

The variance rescaling method (Eqn. 7) shows close agreement between the estimated variance in the MSD from a single simulation and the true numerical variance obtained by sampling many equivalent simulations. The estimated variance varies smoothly with timestep, i. In the example shown, this method overestimates $\sigma^2[x_i]$ at long times, which we attribute the the relatively small number of observed squared displacements that are used to estimate $\sigma^2[x_i]$ in the large i regime.

In comparison, the block renormalisation method also provides reasonable estimates of the true numerically determined variance, but is generally less accurate than

the variance rescaling method. This method exhibits more noise, with larger scatter in $\hat{\sigma}^2[x_i]$ as the timestep i changes. This greater noise is not entirely surprising, since the block renormalisation method aims to independently estimate the correlation length of the input data numerically for each timestep i, while the variance rescaling method takes advantage of the known correlation length due to the way the MSD is computed (Fig. S-1). The block renormalisation method also tends to underestimate $\sigma^2[x_i]$, which can be attributed to its provision of an estimated lower bound for the variance of the mean of the input data (see Eqn. S-35).

In Fig. S-4 we compare the results of estimating D^* and $\sigma^2[\widehat{D}^*]$ using our approximate Bayesian regression scheme, using a model covariance matrix, $\mathbf{\Sigma}'$, as defined in Eqn. 6 in the main text, parameterised by $\hat{\sigma}^2[x_i]$ computed by either variance rescaling or block renormalisation.

Both methods give unbiased estimates of D^* and similar distributions $p(\widehat{D}^*)$. However, the distribution $p(\widehat{D}^*)$ obtained using variance rescaling is slightly narrower than that obtained using block renormalisation. This difference stems from the greater stochastic noise in $\hat{\sigma}^2[x_i]$ when using the block renormalisation method. Consequently, the model covariance matrices, $\mathbf{\Sigma}'$, parametrised from these estimates are themselves noisier and often difficult to condition, leading to numerical instabilities. Both the increased noise and numerical instabilities when conditioning contribute to a wider distribution in $p(\widehat{D}^*)$ when block renormalisation is used to estimate $\sigma^2[x_i]$.

The choice of method used to estimate $\sigma^2[x_i]$ also influences the estimate of the uncertainty in $\widehat{D}^*$. The variance rescaling method provides a good estimate for $\sigma^2[\widehat{D}^*]$ but systematically overestimate the true uncertainty. In contrast, the block renormalisation method provides a similarly good estimate but systematically *underestimates* the true uncertainty in $\widehat{D}^*$. When estimating D^* from molecular dynamics simulations, we consider overestimation of $\sigma^2[\widehat{D}^*]$ to always be preferable to underestimation. Overestimated uncertainty in $\widehat{D}^*$ can be addressed by collecting more data, for example, by performing longer simulations. Conversely, underestimated uncertainty in $\widehat{D}^*$ may lead to false confidence in the accuracy of D^* estimates, which can potentially lead to downstream errors in inferential reasoning or formal hypothesis testing.

S-III: Bias in $p(\hat{\sigma}^2[\widehat{D}^*])$

In the main manuscript, we present results for a set of 4096 3D-lattice random walk simulations, each consisting of 128 particles undergoing 128 steps (Fig. 4). Our approximate Bayesian regression scheme allows us to estimate the variance in $\widehat{D}^*$, denoted as $\hat{\sigma}^2[\widehat{D}^*]$, that would be obtained over a large number of repeat simulations. This estimate is calculated from the variance of the marginal posterior distribution $p(D^*|\boldsymbol{m})$, which we derive from analysis of a single simulation trajectory. As shown in Fig. 4d, our estimate for the population variance $\sigma^2[\widehat{D}^*]$, obtained from a single simulation, aligns reasonably with the true value. When considering the distribution of estimated variance, $p(\hat{\sigma}^2[\widehat{D}^*])$, however, we observe a systematic overestimation (bias) relative to the true value.

This bias arises from our use of estimated variances $\hat{\sigma}^2[x_i]$ when parametrising the model covariance matrix $\mathbf{\Sigma}'$. Figure S-5 presents equivalent results for $p(\widehat{D}^*)$ and $p(\hat{\sigma}^2[\widehat{D}^*])$ for the same 4096 individual simulations, but calculated using a numerical covariance matrix, $\mathbf{\Sigma}_{\text{num}}$ derived from all 4096 observed MSDs. The resulting distribution $p(\hat{\sigma}^2[\widehat{D}^*])$ (see Fig. S-5b) is unbiased. Furthermore, the distribution $p(\widehat{D}^*)$ agrees even more closely with the numerically converged distribution obtained when combining data from all 4096 simulations (Fig S-5a), contrasting with the results presented in Fig. 4b, where our approximate Bayesian regression scheme yields a slightly broadened distribution due to the use of the long-time limit in the derivation of the analytical form for $\mathbf{\Sigma}'$.

S-IV: Comparison of OLS, WLS, and GLS used to estimate D^* in $Li_7La_3Zr_2O_{12}$

In the main manuscript, Fig. 1 shows example distributions of estimated self-diffusion coefficients, $\widehat{D}^*$, calculated using OLS, WLS, and GLS estimators from MSD data from 4096 3D lattice random walk simulations. This figure shows that GLS gives a much narrower distribution of $\widehat{D}^*$ than either OLS or WLS, and also allows the width of this distribution (characterised by $\sigma^2[\widehat{D}^*]$) to be accurately estimated, in contrast with OLS and WLS, which both give estimates of $\sigma^2[\widehat{D}^*]$ that significantly underestimate the true variance.

While the 3D lattice random walk represents an idealised model system, OLS and WLS show the same deficiencies when used to estimate D^* from MSD data from simulations of more complex "real world" systems. Fig. S-6 shows an equivalent comparison between OLS, WLS, and GLS applied to simulation data for the lithium solid electrolyte, $Li_7La_3Zr_2O_{12}$ (LLZO). As for the idealised 3D lattice random walk data, OLS and WLS both give wider distributions of estimated diffusion coefficients, $p(\widehat{D}^*)$, while also systematically underestimating the uncertainty in these estimates. In contrast, GLS gives a narrower distribution of estimated values, and accurately estimates this uncertainty from single simulation data.

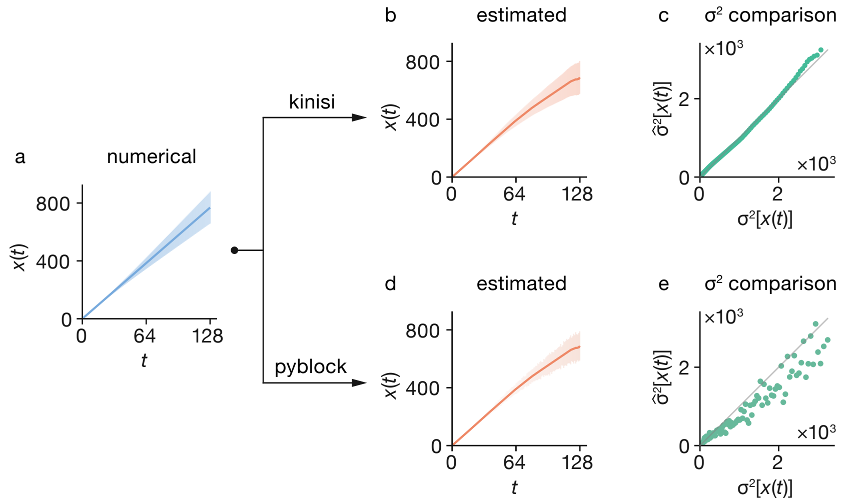

FIG. S-3. Comparison of the numerical variance in observed MSD from multiple replica simulations (panel a), the estimated variance obtained by rescaling the variance in observed squared displacements from a single simulation (panels b and c) (Eqn. 7), and the estimated variance obtained from the block renormalisation method of Flyvbjerg and Peterson [1], as implemented in PYBLOCK [8]. Panel (a) shows the mean observed MSD from 4096 simulations of 128 particles undergoing a 3D lattice random walk of 128 steps per particle, with error bars of $\pm 2\sigma^2[x_i]$. Panel (b) shows the MSD from just one simulation, with error bars of $\pm\widehat{\sigma}^2[x_i]$, obtained via Eqn. 7. Panel (d) shows the same one-simulation MSD, again with error bars of $\pm\widehat{\sigma}^2[x_i]$, obtained using the block renormalisation method of Flyvbjerg and Peterson [1]. Panels (c) and (e) plot the numerical variance against the single-simulation estimated variances obtained with each method, as a function of timestep, i.

[1] Flyvbjerg, H. & Petersen, H. G. Error estimates on averages of correlated data. *J. Chem. Phys.* **91**, 461–466 (1989).

[2] McCluskey, A. R., Coles, S. W. & Morgan, B. J. msd-errors-1.0.0. https://github.com/arm61/msd-errors (2024).

[3] Coles, S. W. Molecular dynamics simulations of cubic llzo at 700 K (2024). URL `https://doi.org/10.5281/zenodo.10532134`.

[4] Smith, W. & Gillan, M. J. The Random Walk and the Mean Squared Displacement. *Inf. Q. Comput. Simul. Condens. Phases* 54–64 (1996).

[5] Howard, R. E. & Lidiard, A. B. Matter transport in solids. *Rep. Prog. Phys.* **27**, 161–240 (1964).

[6] Pranami, G. & Lamm, M. H. Estimating error in diffusion coefficients derived from molecular dynamics simulations. *J. Chem. Theory Comput.* **11**, 4586–4592 (2015).

[7] Frenkel, D. & Smit, B. *Understanding Molecular Simulation* (Academic Press, San Diego, CA, 2023), 3 edn.

[8] Spencer, J., Eikås, R. D. R., Neufeld, V. & Poole, T. pyblock-0.6. https://github.com/jsspencer/pyblock (2020).

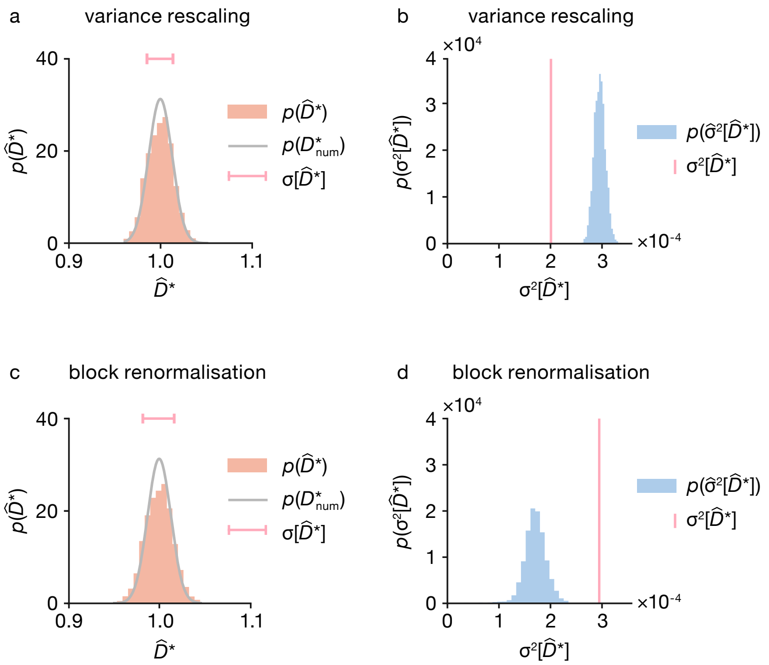

FIG. S-4. (Panels (a) and (c)) Probability distributions of point-estimates $p(D^*)$ obtained from 4096 individual random-walk simulations. Panel (a) shows data obtained using variance rescaling to estimated the variances, $\widehat{\sigma}^2[x_i]$, used to parametrise the model covariance matrix, $\mathbf{\Sigma}'$. Panel (b) shows data obtained using block renormalisation to estimate the variances, $\widehat{\sigma}^2[x_i]$, used to parametrise the model covariance matrix. The grey lines show the distribution of point estimates, $p(\widehat{D}^*)$, obtained using Bayesian regression with a mean vector and numerical covariance matrix derived from the complete dataset of all 4096 simulations. The pink horizontal bar shows an interval of one standard deviation in $p(\widehat{D}^*)$. Panels (b) and (d) show the corresponding probability distributions of estimated variances, $\widehat{\sigma}^2[\widehat{D}^*]$, for individual random-walk simulations, compared to the true sample variances obtained using each method (pink vertical lines) $\sigma^2[\widehat{D}^*]$.

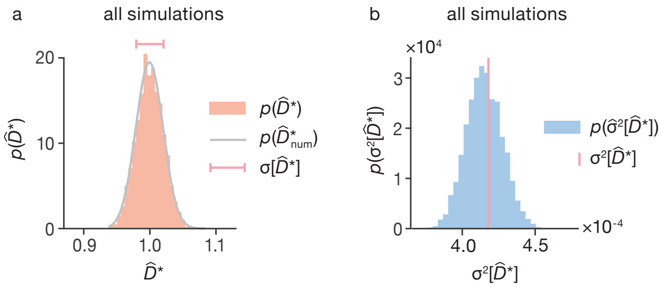

FIG. S-5. (a) Probability distribution of point-estimates, $p(\widehat{D}^*)$, obtained from 4096 individual random-walk simulations, using the numerical covariance matrix $\mathbf{\Sigma}_{\text{num}}$. Each simulation has been analysed as in Fig. 4(a) and (b) to yield a single corresponding point estimate $\widehat{D}^*$. The grey line shows the distribution of point estimates, $p(\widehat{D}^*_{\text{num}})$, obtained using Bayesian regression with a mean vector and numerical covariance matrix derived from the complete dataset of all 4096 simulations. The pink horizontal bar shows an interval of one standard deviation in $p(\widehat{D}^*)$. (b) Probability distribution of estimated variances, $\widehat{\sigma}^2[\widehat{D}^*]$, for individual random-walk simulations, using the numerical covariance matrix $\mathbf{\Sigma}_{\text{num}}$, compared to the true sample variance (pink vertical line) $\sigma^2[\widehat{D}^*]$.

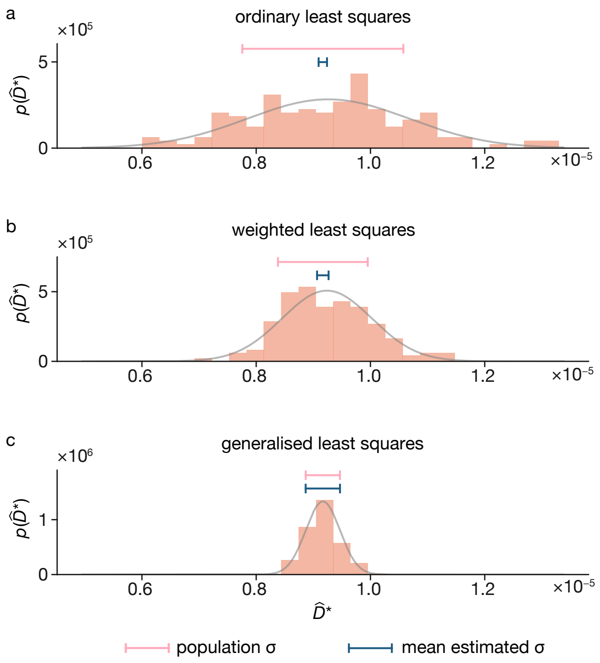

FIG. S-6. Example distributions of estimated self-diffusion coefficients, $\widehat{D}^*$, calculated using (a) ordinary least squares (OLS), (b) weighted least squares (WLS), and (c) generalised least squares (GLS), from MSD data from 512 effective simulations of LLZO of $\sim$25 ps with 56 lithium ions. In each panel, the grey curve shows the best-fit normal distribution for the simulation data, the upper horizontal bar shows the standard deviation of this distribution, and the lower horizontal bar shows the average estimated standard distribution given by the analytical expression for $\sigma^2[\widehat{D}^*]$ for each regression method.
